# Impact of dry eye disease on psychological symptoms among Chinese doctoral students studying abroad

**DOI:** 10.1097/MD.0000000000039786

**Published:** 2024-09-20

**Authors:** Lei Sun, Tao Wang, Jie Gao, Gaoyuan Yang

**Affiliations:** a Department of Ophthalmology, Linyi People’s Hospital, Linyi, China; b Linyi Health School of Shandong Province, Linyi, China; c School of Clinical Medicine, Shandong Second Medical University, Weifang, China.

**Keywords:** depression, dry eye disease, psychology, stress, student

## Abstract

This study aimed to evaluate the impact of dry eye disease (DED) on depression, anxiety, and stress among Chinese doctoral students studying abroad. This is a cross-sectional study. This study enrolled 185 Chinese doctoral students pursuing education in the Philippines. DED was assessed using the Ocular Surface Disease Index, while psychological symptoms were evaluated using the abbreviated version of the Depression, Anxiety, and Stress Scale-21 questionnaire. A survey encompassing demographic information, potential DED risk factors, and individual habits was also administered. Of the 185 students, 129 completed the survey, of which 40 (31.0%) were male and 89 (69.0%) were female. The average age was 36.3 ± 7.0 (mean ± SD; range, 22–57) years. The prevalence of DED, depression, anxiety, and stress was 73.6% (95/129), 43.4% (56/129), 50.4% (65/129), and 22.5% (29/129), respectively. Univariate analysis revealed that aging (*P* < .001), prolonged visual display terminal (VDT) use (*P* = .004), extended paperwork time (*P* < .001), higher depression score (*P* = .006), higher anxiety score (*P* < .001), and higher stress score (*P* < .001) were associated with increased influence of DED. After adjusting for age, duration of VDT use, duration of paperwork, and depression score, age (*P* = .030) had significant association with DED. Additionally, after adjusting for age, duration of VDT use, duration of paperwork, and anxiety score, age (*P* = .026) and anxiety score (*P* = .047) were significantly associated with DED. Moreover, after adjusting for age, duration of VDT use, duration of paperwork, and stress score, age (*P* = .035) and stress score (*P* = .028) showed significant associations with DED. In the multivariate analysis of variance, there was a significant impact of DED severity classification on psychological distress (V = 0.19, F_(9, 375)_ = 2.83, *P* = .003). Univariate analysis of variances indicated that DED severity had a significant impact on anxiety F_(3, 125)_ = 6.06, *P* = .001 and stress F_(3, 125)_ = 3.00, *P* = .033. A higher influence of DED was related to stress and anxiety. Anxiety and stress levels increase with the severity of DED.

## 1. Introduction

Dry eye disease (DED) is a global condition characterized by an imbalance in the tear film, resulting from either insufficient tear production or excessive evaporation of tears.^[1]^ The prevalence of DED worldwide ranges from 9.0% to 29.5%,^[2]^ while the pooled prevalence of DED in China was found to be 17.0%.^[3]^ Furthermore, a recent cross-sectional study investigating the prevalence of DED among Chinese high school students revealed that the prevalence rate of DED reached 70.5%.^[4]^ It has become a major public health problem because of the considerable economic burden it imposes on affected individuals and society.^[5]^ The eye discomfort, pain, redness, dryness, foreign body sensation, and visual disturbances of DED can interfere with daily activities, including reading, driving, and using video terminals; as a result, the disease can seriously impair quality of life.^[6]^ DED has been found to be associated with depression, stress, and anxiety, making it an eye disease that can contribute to frailty.^[7,8]^ And it has been shown that subjective DED symptoms can be caused by an individual’s perception of pain or psychosomatic conditions such as depression, anxiety, and stress.^[9]^ Additionally, a study has shown that the severity of DED symptoms affects psychosomatic symptoms and quality of life in healthy youth groups.^[10]^ It was found that the severity of DED symptoms has a greater impact on depressive symptoms compared to other psychosomatic symptoms.^[10]^ Furthermore, DED has been reported to share a genetic susceptibility to depression.^[11]^

Currently, there is a growing interest in understanding the relationship between DED and psychological disorders. Previous studies have primarily focused on hospital patients, military personnel, hospital staff, and even firefighters. However, to the best of our knowledge, the prevalence of DED and its association with psychological health among Chinese doctoral students studying abroad is unknown. International students are often exposed to the large amount of academic competition and life pressure. Additionally, prolonged use of video terminals and frequent late-night activities further increase the likelihood of developing DED and psychological disorders. Therefore, in this study, we investigated the epidemiological landscape of DED and psychological disorders in international students, focusing on the impact of DED on psychological disorders.

## 2. Methods

### 2.1. Study design and participants

This study was approved by the institutional review board of the Linyi People’s Hospital (approval no. YX200671) and was conducted in accordance with the tenets of the Declaration of Helsinki. Explicit informed consent was obtained from each participant included in the study.

This cross-sectional study was conducted in June 2023 using random whole cluster sampling method to randomly select a community based in Makati, Manila, Philippines, consisting of 185 doctoral students studying abroad from China. All students have formed a WeChat group through a social media platform (WeChat instant messaging app, Shenzhen, China). The questionnaire (Questionnaire Star app, Changsha, China) was sent and prompted to individuals through WeChat. The nature and purpose of the survey and how to answer the questions were explained in detail. Raosoft calculator (Raosoft, Inc., Washington) was used in getting sample size with a margin of error of 5%, confidence level of 95%, and response distribution of 50%. The calculated sample size and actual sample population were 126 and 129, respectively.

The survey consisted of 4 sections: demographics, potential risk factors for DED, dry eye symptoms, and psychological symptoms. The first and second sections entailed information such as gender (male/female), age (year), contact lens use, refractive surgery, hours of visual display terminal (VDT) use per day, durations of paperwork per day, and smoking habits. This study included only those students who had a complete understanding of the nature of the survey and agreed to participate. Exclusion criteria included infectious keratitis and conjunctivitis, connective tissue disorders, systemic vasculitis, glaucoma, history of ocular trauma, any type of ocular surgery except refractive surgery, and psychological disorders.

### 2.2. Questionnaires

In this study, 2 validated questionnaires were utilized. The Ocular Surface Disease Index (OSDI) was used for assessing dry eye symptoms that were experienced during the week before the interview. The OSDI consists of a 12-item questionnaire designed to assess the severity of dry eye according to a score that ranges from 0 to 100. The total OSDI score was computed for each participant using the following formula: sum of scores of all questions answered multiplied by 25 divided by the total number of questions answered. Participants were classified into 4 groups by OSDI score: normal (0–12 scores), mild (13–22), moderate (23–32), and severe (33–100). Symptomatic DED is defined as an OSDI score above 12.^[12,13]^

The 21-item Depression Anxiety Stress Scale-21 (DASS-21) was used in measuring psychological distress in this study. The DASS-21 consists of 3 separate scales, each comprising 7 items.^[14]^ Participants were instructed to carefully read each statement and indicate how each item applied to them over the past week. Response options ranged from 0 (never) to 3 (always). The responses for each scale were summed and multiplied by 2 to obtain the total score for that particular subscale. Based on the total score, participants were categorized into 5 levels of psychological distress: normal, depression (≤9 scores), anxiety (≤7 scores), and stress (≤14 scores); mildly, depression (10–13 scores), anxiety (8–9 scores), and stress (15–18 scores); moderately, depression (14–20), anxiety (10–14), and stress (19–25); severe, depression (21–27), anxiety (15–19), and stress (26–33); and extremely severe, depression (≥28), anxiety (≥20), and stress (≥34).^[14,15]^

### 2.3. Statistical analysis

All analyses were performed using SPSS software version 26.0 (IBM Corporation, Armonk). The prevalence of DED, depression, anxiety, and stress was estimated with 95% confidence intervals (CI). Data are presented as mean ± standard deviation. Independent samples *t* test for quantitative variables and Pearson chi-square test for categorical variables were employed to evaluate the relationship between potential influencing factors and DED. Multivariate analysis was performed using logistic regression analysis to identify the influencing factors of DED. Multivariate analysis of variance (MANOVA) was utilized to assess potential variations in DASS-21 scores across the 3 subsections concerning increasing dry eye severity, as classified by the OSDI designation for dry eye severity. In the event of a statistically significant MANOVA outcome, subsequent univariate analyses of variances were conducted for each of the dependent variables. A *P* value < .05 was taken as statistically significant.

## 3. Results

Among all 185 doctoral students, 129 completed the survey, of which 40 (31.0%) were male and 89 (69.0%) were female. The average age was 36.3 ± 7.0 (mean ± SD; range, 22–57) years. The prevalence of DED, depression, anxiety, and stress were 73.6% (95/129; 95% CI, 65.9–81.3%), 43.4% (56/129; 95% CI, 34.7%–52.1%), 50.4% (65/129; 95% CI, 41.6%–59.1%), and 22.5% (29/129; 95% CI, 15.2%–29.8%), respectively. Table 1 shows the mean values and standard deviations of the scores for the OSDI and DASS-21 subscales based on the OSDI classification of severity.

**Table 1:** Mean values and SDs of OSDI and DASS-21 subscale scores based on OSDI classification of severity.

Severity of dry eye	OSDI score	Depression subscale	Anxiety subscale	Stress subscale
Normal				
N	34	34	34	34
M ± SD	4.41 ± 3.71	6.12 ± 6.32	6.41 ± 5.88	8.00 ± 6.62
Mild				
N	34	34	34	34
M ± SD	16.88 ± 2.95	7.65 ± 6.61	6.53 ± 6.12	10.35 ± 6.92
Moderate				
N	25	25	25	25
M ± SD	25.72 ± 2.51	9.04 ± 5.45	10.48 ± 6.72	12.00 ± 5.45
Severe				
N	36	36	36	36
M ± SD	52.00 ± 15.78	10.33 ± 7.41	11.83 ± 7.26	12.39 ± 7.10
Total				
N	129	129	129	129
M ± SD	25.11 ± 20.25	8.26 ± 6.70	8.74 ± 6.89	10.62 ± 6.79

DASS *=* depression anxiety stress scale, M *=* mean, N *=* number of participants, OSDI *=* ocular surface disease index, SDs *=* standard deviations.

The results of univariate analysis revealed that aging (*P* < .001), prolonged VDT use (*P* = .004), extended paperwork time (*P* < .001), higher depression score (*P* = .006), higher anxiety score (*P* < .001), and higher stress score (*P* < .001) were associated with increased influence of DED. Compared to non-DED group, the DED group showed older age (37.29 ± 6.97vs 32.59 ± 7.99 years, *P* < .001), longer duration of VDT use (8.29 ± 3.55vs 7.68 ± 3.42 hours, *P* = .004), and longer time of paper work (4.1 ± 3.07vs 3.68 ± 2.83 hours, *P* < .001). Furthermore, depression score (9.03 ± 6.69 vs 6.12 ± 6.32, *P* = .006), anxiety score (9.58 ± 7.06 vs 6.41 ± 5.88, *P* < .001), and stress score (11.56 ± 6.63 vs 8.00 ± 6.22, *P* < .001) were significantly higher in DED group. However, sex, contact lens use, history of refractive surgery, or smoking did not show a significant association with the risk of DED (Table 2).

**Table 2:** Univariate analysis of potential influencing factors for DED.

Characteristic	Non-DED group (n = 34)	DED group (n = 95)	*t* (*χ*^2^)	*P*
Age (yr)	32.59 ± 7.99	37.29 ± 6.97	6.012	<.001
Sex (male/female)	11/23 (32.35%/67.65%)	29/66 (30.53%/69.47%)	0.039*	.843
VDT use (h)	7.68 ± 3.42	8.29 ± 3.55	2.883	.004
Paper work (h)	3.68 ± 2.83	4.13 ± 3.07	4.267	<.001
Contact lens use	23.53% (8/34)	33.68% (32/95)	1.207*	.272
History of refractive surgery	5.88% (2/34)	5.26% (5/95)	0.019*	.891
Smoking	11.76% (4/34)	5.26% (5/95)	1.631*	.202
Depression score	6.12 ± 6.32	9.03 ± 6.69	2.753	.006
Anxiety score	6.41 ± 5.88	9.58 ± 7.06	4.890	<.001
Stress score	8.00 ± 6.22	11.56 ± 6.63	3.658	<.001

The data are presented as mean ± SD, or percentage.

DED group *=* participants with dry eye disease, Non-DED group *=* participants without dry eye disease, VDT *=* visual display terminal.

* The value was determined through the utilization of Pearson chi-square test.

In Table 3, the multivariate logistic regression analyses were conducted using 3 models. After adjusting for age, duration of VDT use, duration of paperwork, and depression score, age (odds ratio [OR], 1.071; 95% CI, 1.006–1.139; *P* = .030) had a significant association with DED (model 1). Additionally, after adjusting for age, duration of VDT use, duration of paperwork, and anxiety score, age (OR, 1.073; 95% CI, 1.009–1.142; *P* = .026) and anxiety score (OR, 1.074; 95% CI, 1.001–1.152; *P* = .047) were significantly associated with DED (model 2). Moreover, after adjusting for age, duration of VDT use, duration of paperwork, and stress score, age (OR, 1.070; 95% CI, 1.005–1.139; *P* = .035) and stress score (OR, 1.078; 95% CI, 1.008–1.153; *P* = .028) showed significant associations with DED (model 3).

**Table 3:** Multivariate logistic regression analysis of potential influencing factors for DED.

Characteristic	Model 1		Model 2		Model 3	
	OR (95% CI)	*P*	OR (95% CI)	*P*	OR (95% CI)	*P*
Age (yr)	1.071 (1.006–1.139)	.030	1.073 (1.009–1.142)	.026	1.070 (1.005–1.139)	.035
VDT use (h)	0.950 (0.843–1.071)	.402	0.951 (0.843–1.072)	.410	0.949 (0.840–1.071)	.393
Paper work (h)	1.041 (0.901–1.203)	.586	1.039 (0.900–1.201)	.598	1.029 (0.891–1.187)	.699
Depression score	1.068 (0.995–1.147)	.069	-	-	-	-
Anxiety score	-	-	1.074 (1.001–1.152)	.047	-	-
Stress score	-	-	-	-	1.078 (1.008–1.153)	.028

Model 1: Adjusted for age, duration of VDT use, duration of paperwork, and depression score. Model 2: Adjusted for age, duration of VDT use, duration of paperwork, and anxiety score. Model 3: Adjusted for age, duration of VDT use, duration of paperwork, and stress score. - Represents no value.

CI *=* confidence interval, DED *=* dry eye disease, OR *=* odds ratio, VDT *=* visual display terminal.

According to the analysis utilizing Pillai trace in the MANOVA, there was a significant impact of DED severity classification on psychological distress (V = 0.19, F_(9, 375)_ = 2.83, *P* = .003). Univariate analysis of variances indicated that DED severity had a significant impact on anxiety F_(3, 125)_ = 6.06, *P* = .001 and stress F_(3, 125)_ = 3.00, *P* = .033 but did not show a significant influence on depression F_(3, 125)_ = 2.61, *P* = .054. Post hoc analysis was conducted using the LSD test of multiple comparison to examine variations in psychological distress across different severity groups of DED. For the depression scores, there were no significant differences between 4 categories of DED (*P* > .05) except the normal group and severe DED group (*P* = .008). There were statistically significant differences in anxiety scores between the normal and mild DED groups compared to the moderate and severe DED groups, respectively (*P* < .05), while no significant differences were observed among the other groups (*P* > .05). In addition, the stress score of the normal group was significantly different from that of the moderate and severe DED groups (*P* < .05), but there was no significant difference among other groups (*P* > .05).

## 4. Discussion

In this study, we evaluated the prevalence and potential factors of DED in Chinese doctoral students studying in the Philippines. Symptomatic DED has been employed to analyze the prevalence and potential factors associated with DED worldwide, particularly in large population-based researches.^[16–18]^ When compared to other epidemiological studies on DED that utilized the same diagnostic criteria through OSDI questionnaires, a separate investigation concentrating on DED prevalence among high school students in Mexico uncovered that 65.3% of these students experienced symptomatic DED.^[19]^ The prevalence of symptomatic DED among medical students in Korea and Chinese high school students was 27.1% and 70.5%, respectively.^[4,20]^ Another study based on OSDI questionnaires showed a prevalence of symptomatic DED of 44.3% among university undergraduate students in Ghana.^[16]^ In a cross-sectional study involving Brazilian university students, it was found that 59.6% of participants registered scores exceeding 12 on OSDI questionnaires.^[21]^ The prevalence reported in our study was higher than that of the 5 studies mentioned above. Considering the high prevalence of DED among Chinese doctoral students studying in the Philippines in this study, we speculate that this may be related to environmental changes, life, and study pressure. On the one hand, studying abroad can mean moving from 1 climate and environment to another, which can have an effect on the eyes. On the other hand, international students usually need to adapt to a new lifestyle and may face stress, lack of sleep, and poor eating habits, all of which can affect eye health. Finally, international doctoral students are under a lot of pressure to study, especially to finish their graduation thesis on time, which often requires prolonged use of computers or mobile phones, which can lead to eye symptoms.

This study showed that aging was related to DED, aligning harmoniously with the outcomes of the previous studies.^[22,23]^ Nonetheless, the debate persists regarding whether aging directly triggers dry eye or if dry eye represents an age-related ailment governed by mechanisms distinct from the aging process itself.^[24]^ Age-related dry eye is progressively understood to involve substantial inflammation and complex immune responses, which ultimately lead to significant changes in both the lacrimal gland and the ocular surface.^[25,26]^ Gaining insights into the aging mechanisms holds the potential to facilitate early interventions and preemptive measures against ocular surface diseases.^[27]^

In the univariate analysis of this study, prolonged VDT use and extended paper work time were associated with increased influence of DED, but such associations did not maintain significance in the multivariate analysis. Previous studies have shown that the prolonged use of VDT was a risk factor related to DED.^[18,28]^ The prevailing hypothesis suggests that the usage of VDT has an impact on blinking patterns, leading to reduced blink frequency and incomplete blinks, which in turn contribute to heightened dryness of the ocular surface. Notably, while digital screen reading has been linked to decreased blink rates, a similar effect has been observed when reading from a traditional book, indicating that electronic screen exposure is not the sole determinant of this phenomenon.^[29]^ Given the increasing prevalence of iPads, iPhones, and laptops, it is imperative to underscore the elevated potential for prolonged VDT use engagement to trigger DED.^[30]^

To the best of our knowledge, this study is the first to reveal the relationship between psychological symptoms and DED symptoms among Chinese doctoral students studying abroad. This study showed that anxiety and stress were associated with an increased influence of DED. Depression was also related to DED in univariate analysis, although no significant relationship was found in multivariate logistic regression analysis. There was a significant impact of the DED severity classification on psychological distress in the MANOVA. Univariate analysis of variances indicated that DED severity had a significant impact on anxiety and stress but did not show a significant influence on depression. This discovery aligns with numerous other investigations that have demonstrated a connection between psychological symptoms and DED symptoms.^[7,8,20]^ In addition to depression, this study confirmed that the more severe the dry eye, the more obvious the anxiety and stress. The relationship between DED and psychological symptoms may be attributed to various mechanisms, although a comprehensive understanding of these mechanisms is still lacking. Psychological stress has been identified as a potential factor that influences pain perception. Stressors can trigger an increase in cortisol levels, which in turn can amplify the experience of pain.^[31]^ Additionally, acute stress has been observed to diminish an individual’s capacity to modulate pain effectively.^[32]^ Consequently, it is plausible to consider that individuals experiencing heightened psychological stress may exhibit an increased susceptibility to dry eye symptoms.^[20]^ Furthermore, it has been proposed that chronic depression may contribute to the development of dry eye symptoms by fostering the production and release of proinflammatory cytokines within the body.^[33,34]^ A commonly reported phenomenon in depression known as somatization may also play a role in precipitating the onset of dry eye symptoms. Conversely, the presence of dry eye symptoms, characterized by visual disturbances and persistent discomfort, can potentially exacerbate depressive moods and induce feelings of anxiety.^[7]^ Notably, pain and visual blurring resulting from tear film instability have been suggested as factors that can predispose individuals with dry eyes to experience heightened tendencies toward depression, anxiety, and stress.^[34,35]^ In addition, this study proposes 3 potential mechanisms that may offer insights into the connection between DED and anxiety and stress: climate and environmental variations, cultural disparities, and demanding academic standards in foreign nations.

The present study has several limitations. First, the relatively modest sample size may not be entirely representative of the entire population of Chinese doctoral students studying abroad, and the overestimation of DED prevalence is possible, given that all participants were doctoral students engaged in prolonged VDT use. Nonetheless, this study contributes valuable insights into the nature of DED among doctoral students, particularly by demonstrating an association with psychological stress and anxiety. Future investigations should encompass larger and more diverse participant cohorts to enhance our understanding of DED characteristics in this demographic. Second, our study lacks objective measures of dry eye signs, and the prevalence of DED could potentially vary depending on the adopted diagnostic criteria. This study reports only the prevalence of symptomatic DED based on the OSDI questionnaire as a potential explanation for the high frequency of reported DED. Subsequent research endeavors should integrate objective dry eye assessments to corroborate the findings derived from symptom-based questionnaires. Third, the present data relies on self-report scales, which may introduce inherent subjectivity and potential biases into the responses obtained.

In conclusion, the study found that anxiety and stress were associated with an increased influence of DED in Chinese doctoral students studying in the Philippines. Depression was found to be associated with DED. The greater the severity of DED, the more apparent the presence of anxiety and stress.

## Author contributions

**Conceptualization:** Lei Sun, Gaoyuan Yang.

**Data curation:** Lei Sun, Jie Gao, Gaoyuan Yang.

**Formal analysis:** Lei Sun, Tao Wang.

**Methodology:** Lei Sun, Gaoyuan Yang.

**Resources:** Lei Sun, Tao Wang, Jie Gao, Gaoyuan Yang.

**Software:** Lei Sun.

**Writing – original draft:** Lei Sun.

**Investigation:** Tao Wang, Jie Gao, Gaoyuan Yang.

**Project administration:** Tao Wang, Gaoyuan Yang.

**Supervision:** Tao Wang, Gaoyuan Yang.

**Validation:** Tao Wang, Jie Gao, Gaoyuan Yang.

**Writing – review & editing:** Tao Wang, Jie Gao, Gaoyuan Yang.
